# Mortality from vertebral fractures in White women aged 65+ with osteoporosis: a CDC database trend analysis from 1999 to 2020

**DOI:** 10.1093/jbmrpl/ziaf121

**Published:** 2025-07-18

**Authors:** Muhammad Hammad Zaheer, Hamza Zaheer, Arslan Tariq

**Affiliations:** Allama Iqbal Medical College, Lahore, Punjab 54700, Pakistan; Services Institute of Medical Sciences, Lahore, Punjab 54000, Pakistan; Kyrgyz State Medical Academy, Bishkek, Kyrgyzstan

**Keywords:** osteoporosis, vertebral fractures, mortality trends, bone health, joinpoint regression, epidemiology

## Abstract

Osteoporotic vertebral fractures represent a significant yet underdiagnosed manifestation of osteoporosis, particularly affecting older White women. While vertebral fractures are among the most common osteoporotic fractures, their contribution to mortality has received less attention compared to hip fractures, creating a critical knowledge gap. This study analyzed temporal trends in age-adjusted mortality rates from osteoporotic vertebral fractures among White women aged 65 and older in the United States from 1999 to 2020. We conducted a retrospective analysis using Centers for Disease Control and Prevention’s Multiple Cause of Death files, identifying cases where both vertebral fractures and osteoporosis were listed as causes of death using specific ICD-10 codes. Age-adjusted mortality rates per 100 000 population were calculated using the 2000 US standard population, and joinpoint regression analysis identified significant changes in mortality trends over the 22-yr study period. Our findings revealed a concerning 87.5% increase in age-adjusted mortality rates, rising from 0.24 per 100 000 in 1999 to 0.45 per 100 000 in 2020. Joinpoint regression identified three distinct trend segments: a non-significant decline from 1999 to 2004, followed by a statistically significant increase from 2004 to 2009 with an annual percent change (APC) of 13.93%, and a more modest upward trend from 2009 to 2020. The overall average APC was 4.21%, indicating a highly significant upward trend in mortality rates. The pronounced increase during 2004-2009 coincides with important developments in osteoporosis management, including declining hormone replacement therapy use following Women’s Health Initiative findings and emerging bisphosphonate safety concerns. These findings underscore vertebral fractures as potentially life-threatening complications requiring aggressive prevention and management strategies. As the population ages, our results highlight the urgent need for improved osteoporosis screening, enhanced fracture risk assessment, and optimized treatment approaches to reduce the growing burden of vertebral fracture-related mortality in this vulnerable population.

## Graphical Abstract

**Graphical Abstract:**
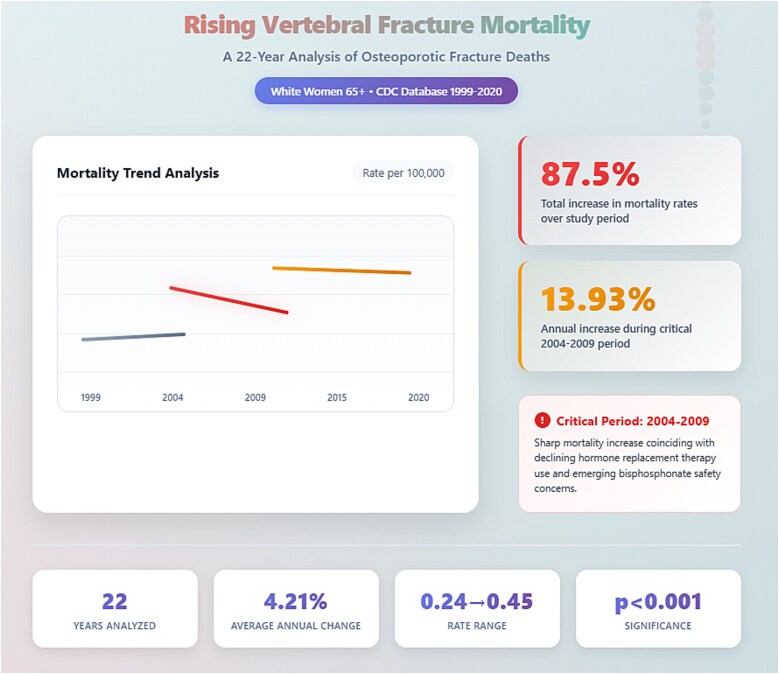

## Introduction

Osteoporosis represents a significant public health challenge, particularly among older White women who bear a disproportionate burden of this disease. This skeletal disorder is characterized by compromised bone strength, predisposing individuals to an increased risk of fractures, with vertebral fractures being among the most common yet underdiagnosed manifestations of osteoporosis.^1^ Despite their prevalence, vertebral fractures have received less attention in mortality research compared to hip fractures, creating a critical knowledge gap in our understanding of osteoporosis-related mortality.

Vertebral fractures significantly impact quality of life and are associated with substantial morbidity, including chronic pain, height loss, kyphosis, reduced pulmonary function, and impaired mobility.^2^ Recent evidence suggests that these fractures may also contribute to excess mortality, though the temporal trends in mortality specifically attributed to osteoporotic vertebral fractures have not been comprehensively examined in the United States.^3^

The aging of the population has important implications for the burden of osteoporosis and its complications. With the proportion of Americans aged 65 and older projected to increase substantially over the coming decades, understanding trends in osteoporosis-related mortality becomes increasingly important for healthcare planning and resource allocation.^4^ White women aged 65 and older represent a particularly vulnerable population, with estimated osteoporosis prevalence exceeding 30% in this demographic group.^5^

Previous research has demonstrated changing patterns in osteoporosis management over the past 2 decades, including fluctuations in screening rates, medication use, and clinical guideline implementation.^6^ These shifts in clinical practice may have influenced mortality outcomes, yet their impact specifically on vertebral fracture mortality remains unclear. Additionally, the introduction and subsequent safety concerns regarding various osteoporosis medications during this period may have affected treatment patterns and, consequently, fracture outcomes.^7^

While this study focused on White women due to their high prevalence of osteoporosis, data availability and reliability for other racial groups in CDC’s mortality database is limited, particularly in cases where both vertebral fractures and osteoporosis are listed concurrently. Furthermore, epidemiological evidence consistently shows that White women are at the highest risk of osteoporotic fractures and related mortality compared to other racial/ethnic groups.^3^^,^^5^ Inclusion of other groups would have introduced greater statistical noise and less reliable trend estimates.

This study aims to analyze trends in age-adjusted mortality rates from osteoporotic vertebral fractures among White women aged 65 and older in the United States from 1999 to 2020 using data from the Centers for Disease Control and Prevention’s (CDC) Multiple Cause of Death (MCD) files. By employing joinpoint regression analysis, we identify significant changes in mortality trends over this 22-yr period and discuss potential factors contributing to these patterns. Understanding these trends is crucial for evaluating the effectiveness of current prevention and treatment strategies and for informing future approaches to reduce the burden of osteoporosis-related mortality.

## Materials and methods

### Data source and study population

We conducted a retrospective analysis using publicly available mortality data from the CDC MCD files (1999-2020). These files contain information from death certificates filed in the United States and include demographic data and causes of death coded according to the International Classification of Diseases, 10th Revision (ICD-10).^8^ The study population comprised White females aged 65 yr and older who had vertebral fractures associated with osteoporosis listed as a cause of death.

The data used in this study are publicly available from the CDC WONDER Online Database, specifically the MCD files (1999-2020), which can be accessed at https://wonder.cdc.gov/mcd.html. No special access or permissions are required to obtain these data.

### Case definition

Cases were defined as deaths in which both vertebral fractures and osteoporosis were listed as causes of death. Vertebral fractures were identified using ICD-10 codes, including S12.0 (fracture of first cervical vertebra), S12.1 (fracture of second cervical vertebra), S12.2 (fracture of other specified cervical vertebra), S12.7 (multiple fractures of cervical spine), S22.0 (fracture of thoracic vertebra), S22.1 (multiple fractures of thoracic spine), S32.0 (fracture of lumbar vertebra), S32.1 (fracture of sacrum), S32.2 (fracture of coccyx), S32.7 (multiple fractures of lumbar spine and pelvis), and S32.8 (fracture of other and unspecified parts of lumbar spine and pelvis). Osteoporosis was identified using ICD-10 codes M80.0-M80.9 (osteoporosis with pathological fracture) and M81.0-M81.9 (osteoporosis without current pathological fracture).

Although earlier years in mortality reporting may have involved transitions from ICD-9 to ICD-10, the CDC MCD database provides consistent coding by recoding all causes of death from 1999 onward into ICD-10. Therefore, ICD-10 coding was used uniformly across the study period. Some additional ICD codes potentially related to spinal pathology (eg, M484, DM485, T089, and DM809C) were not included in this analysis. This is because they are rarely used in mortality reporting and are insufficiently validated for cause-of-death classification in this context. Future studies leveraging linked clinical datasets may be better suited to incorporate such codes.

To ensure specificity and analytical reliability, only deaths that included both a vertebral fracture code and an osteoporosis code were considered. This strict inclusion criterion was applied to minimize misclassification and isolate deaths plausibly linked to osteoporotic vertebral fractures.

### Statistical analysis

Annual age-adjusted mortality rates per 100 000 population were calculated using the direct method of standardization with the 2000 U.S. standard population as the reference. Confidence intervals (95% CI) for these rates were computed using the gamma method, which provides more accurate intervals for rare events.^9^

To identify significant changes in mortality trends over time, we performed joinpoint regression analysis using the Joinpoint Regression Program (version 4.9.0) from the National Cancer Institute.^10^ This statistical methodology identifies points (joinpoints) where linear trends change significantly in magnitude or direction. The program fits the simplest joinpoint model that the data allow, starting with zero joinpoints (a straight line) and testing whether more joinpoints are statistically significant. The program calculates the annual percent change (APC) for each identified trend segment and determines whether the APC is significantly different from zero.

For our analysis, we specified a maximum of 3 joinpoints with a minimum of 4 yr between joinpoints. We used the grid search method and Monte Carlo permutation test for model selection, with overall significance level set at 0.05. The final selected model provided the best fit for the observed trend data, balancing model fit and parsimony. Statistical significance was set at *p* < .05 for all trend analyses.

All statistical analyses were conducted in accordance with CDC guidance on the use of public mortality data, and Institutional Review Board (IRB) approval was not required as the data were de-identified and publicly accessible.

## Results

### Age-adjusted mortality rates

**Figure 1:**
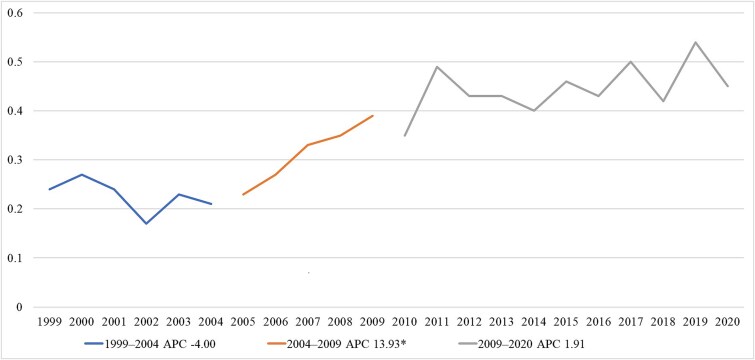
Age-adjusted mortality rates per 100 000 for osteoporotic vertebral fractures in White women aged 65 and older, 1999-2020.

Between 1999 and 2020, age-adjusted mortality rates for osteoporotic vertebral fractures among White women aged 65 and older demonstrated notable variations (Table 1, Figure 1). The mortality rate was 0.24 per 100 000 (95% CI: 0.18-0.32) in 1999, the first year of observation. Over the 22-yr study period, there was an overall increase, reaching 0.45 per 100 000 (95% CI: 0.37-0.53) in 2020, representing an 87.5% increase from the baseline rate.

**Table 1:** Age-adjusted mortality rates per 100 000 for osteoporotic vertebral fractures in White women aged 65 and older, 1999-2020.

**Year**	**Age-adjusted rate**	**95% CI**
**1999**	0.24	0.18-0.32
**2000**	0.27	0.20-0.34
**2001**	0.24	0.18-0.32
**2002**	0.17	0.12-0.23
**2003**	0.23	0.17-0.30
**2004**	0.21	0.15-0.28
**2005**	0.23	0.17-0.30
**2006**	0.27	0.21-0.34
**2007**	0.33	0.25-0.42
**2008**	0.35	0.28-0.44
**2009**	0.39	0.31-0.48
**2010**	0.35	0.28-0.44
**2011**	0.49	0.40-0.58
**2012**	0.43	0.35-0.51
**2013**	0.43	0.35-0.51
**2014**	0.40	0.32-0.48
**2015**	0.46	0.38-0.54
**2016**	0.43	0.34-0.51
**2017**	0.50	0.42-0.58
**2018**	0.42	0.34-0.51
**2019**	0.54	0.45-0.63
**2020**	0.45	0.37-0.53

Rates are age-adjusted using the 2000 US standard population. Data source: CDC Multiple Cause of Death files, ICD-10 codes S22, S32 (spinal fractures) with M80-M82 (osteoporosis).

The lowest mortality rate was observed in 2002 at 0.17 per 100 000 (95% CI: 0.12-0.23), while the highest rate occurred in 2019 at 0.54 per 100 000 (95% CI: 0.45-0.63), more than three times the 2002 rate (Table 1, Figure 1). The data revealed considerable year-to-year fluctuations, though with a general upward trajectory over the entire study period.

After 2009, mortality rates consistently remained above 0.35 per 100 000, whereas before 2007, rates were consistently below this value. The period from 2009 to 2020 showed more variability, with rates oscillating between 0.35 and 0.54 per 100 000, but maintaining a higher baseline compared to the first decade of observation.

### Joinpoint regression

The joinpoint regression analysis identified 3 distinct trend segments over the study period (Figure 1). The first segment, from 1999 to 2004, demonstrated a non-significant APC of −4.00% (95% CI: −12.54%-5.37%, *p* = .363), indicating a slight downward trend that did not reach statistical significance (Table 2).

**Table 2:** Joinpoint regression analysis of mortality trends for osteoporotic vertebral fractures in White women aged 65+, 1999-2020.

**Time period**	**Annual percent change (APC)**	**95% CI**	***p*-value**
**1999-2004**	−4.00	−12.54 to 5.37	.363
**2004-2009**	13.93	0.75 to 28.83	.039^*^
**2009-2020**	1.91	−0.14 to 3.99	.065

^*^Significant at *p* < .05*.* Joinpoint regression analysis performed using model 2. Test statistics were derived from permutation tests.

The second segment, spanning 2004 to 2009, revealed a statistically significant increase in mortality rates with an APC of 13.93% (95% CI: 0.75%-28.83%, *p* = .039). This period represented the most dramatic change in mortality trends over the entire study timeline, with rates nearly doubling from 0.21 per 100 000 in 2004 to 0.39 per 100 000 in 2009.

The third and final segment, from 2009 to 2020, showed a more modest increase with an APC of 1.91% (95% CI: −0.14%-3.99%, *p* = .065). While this upward trend approached statistical significance, it did not meet the threshold of *p* < .05 (Figure 1, Table 2). During this period, mortality rates continued to rise, but at a substantially slower pace compared to the 2004-2009 period.

When considering the entire 22-yr study period (1999-2020), the average APC (AAPC) was 4.21% (95% CI: 3.17%-5.26%, *p* < .001), indicating a statistically significant overall upward trend in mortality rates from osteoporotic vertebral fractures among the study population (Figure 1, Table 2)*.*

## Discussion

This study examined trends in age-adjusted mortality rates from osteoporotic vertebral fractures among White women aged 65 and older in the United States from 1999 to 2020. Our findings reveal a complex pattern of mortality trends characterized by 3 distinct periods: an initial non-significant decline (1999-2004), followed by a significant increase (2004-2009), and then a period of more modest, non-significant growth (2009-2020). The overall trend across the 22-yr period showed a statistically significant increase in mortality rates, with an AAPC of 4.21%.

The pronounced increase in mortality rates during 2004-2009 (APC 13.93%) is particularly noteworthy and warrants careful consideration. This period coincides with several important developments in osteoporosis management that may have influenced mortality outcomes. First, this timeframe follows the 2002 Women’s Health Initiative (WHI) publication that reported increased risks of breast cancer, coronary heart disease, stroke, and pulmonary embolism associated with hormone replacement therapy (HRT).^11^ The subsequent dramatic decline in HRT use may have contributed to increased osteoporosis-related fractures and mortality, as HRT was previously a mainstay of osteoporosis prevention.^12^

Second, the period 2004-2009 also overlaps with the emergence of concerns regarding the safety of bisphosphonates, particularly related to osteonecrosis of the jaw (first reported in 2003-2004) and atypical femoral fractures (gaining attention around 2005-2008).^13^ These safety concerns led to decreased prescription and adherence rates for bisphosphonates, potentially resulting in suboptimal management of osteoporosis in high-risk patients.^14^ The temporal relationship between these medication concerns and the observed increase in vertebral fracture mortality rates suggests a possible causal association that merits further investigation. Furthermore, an increase in screening rates and clinical awareness of osteoporosis during the mid-2000s may have contributed to more frequent diagnosis and death certificate documentation of vertebral fractures. This trend aligns with broader epidemiological patterns, where greater case detection can partially explain rising mortality rates. Such factors may have amplified the observed increase in fracture-related deaths during this period, even in the absence of a true increase in incidence.

The more modest increase in mortality rates during 2009-2020 (APC 1.91%) may reflect an adaptation in clinical practice, including updated treatment guidelines, alternative therapeutic options, and improved fracture risk assessment tools.^15^ Nevertheless, the continued upward trend—though not statistically significant—indicates that vertebral fracture-related mortality remains a persistent clinical challenge. While hip fractures have been more extensively studied and are associated with substantially higher mortality rates—estimated to be over 100 times greater in some studies^16^—they have consequently received far more attention in both research and practice. In contrast, vertebral fractures remain underrecognized and underreported, despite their considerable morbidity and contribution to excess mortality. Our study intentionally focused on vertebral fractures to address this research gap and draw attention to their growing clinical and public health impact. This focus is particularly important given that vertebral fractures often contribute to mortality through indirect mechanisms, including impaired pulmonary function, chronic pain, immobility, and related complications such as pneumonia.^17^

### Limitations

Several important limitations should be acknowledged when interpreting our findings. Our reliance on death certificate data introduces potential for underestimation of mortality burden. Death certificates may not consistently report vertebral fractures as contributing causes of death, particularly when other comorbidities are present. Studies have demonstrated that vertebral fractures are frequently underdiagnosed, with up to two-thirds not coming to clinical attention.^18^ Additionally, while the CDC MCD database is comprehensive, it is primarily based on death certificates and may miss cases where vertebral fractures are not diagnosed or recorded—particularly among patients managed outside of hospital settings. Our case definition requiring concurrent documentation of both vertebral fracture and osteoporosis codes may have further restricted case identification.

The observational nature of our study design precludes drawing causal inferences regarding factors driving the observed mortality trends. While we identified temporal relationships between mortality rates and potential contributing factors, we cannot establish direct causality. Our focus on White women aged 65 and older limits the generalizability of our findings to other demographic groups. We were unable to account for potential confounding factors that might influence mortality outcomes, including comorbidities, medication use, socioeconomic status, and healthcare access.

Changes in ICD-10 coding practices and clinical awareness of osteoporosis over the 2-decade study period may have influenced trend estimates. Increased recognition of osteoporosis as a contributing factor to mortality could lead to more frequent documentation on death certificates, potentially inflating more recent estimates. While our study observed statistically significant increases in mortality rates, the absolute rates remain relatively low (0.45 per 100 000 in 2020). However, given the limitations described regarding case identification, these figures likely represent a substantial underestimation of the true burden of vertebral fracture-related mortality.

### Implications

These findings have important implications for clinical practice and public health policy. The significant increase in mortality rates during 2004-2009 and the continued upward trend thereafter suggest that current approaches to preventing and managing osteoporotic vertebral fractures may be insufficient. Clinicians should maintain vigilance in screening for and treating osteoporosis, particularly in high-risk women, and should not underestimate the potential severity of vertebral fractures. Public health initiatives should focus on increasing awareness of osteoporosis and its complications, improving access to effective treatments, and addressing barriers to optimal care. Additionally, while the CDC MCD database is comprehensive, it relies on death certificate data and may not capture all vertebral fractures, especially those not diagnosed in hospital or emergency settings. This introduces potential underreporting.

Future research should explore these trends in more diverse populations, investigate the factors driving changes in mortality rates, and evaluate the effectiveness of interventions aimed at reducing vertebral fracture incidence and associated mortality. Additionally, studies using linked clinical and administrative data could provide more detailed insights into the pathways from vertebral fracture to mortality and identify opportunities for intervention.

In conclusion, our analysis reveals a concerning trend of increasing mortality from osteoporotic vertebral fractures among older White women in the United States over the past 2 decades, with a particularly sharp rise during 2004-2009. These findings highlight the need for continued efforts to improve osteoporosis management and prevent fracture-related mortality in this vulnerable population.

## Conclusion

This study addressed the critical knowledge gap regarding temporal trends in mortality from osteoporotic vertebral fractures among White women aged 65 and older in the United States. Our analysis revealed a significant 87.5% increase in age-adjusted mortality rates between 1999 and 2020, with a particularly concerning acceleration during the 2004-2009 period (APC 13.93%). Despite their clinical significance, vertebral fractures have historically received less attention than hip fractures in both research and clinical practice. These findings highlight vertebral fractures as potentially life-threatening complications of osteoporosis that warrant aggressive prevention and management strategies. As the population continues to age, our results serve as a call to action for renewed focus on vertebral fracture prevention, improved risk assessment, and optimized treatment approaches to reduce the growing burden of osteoporosis-related mortality in this vulnerable population.
